# High-frequency oscillations and sleep spindles in epilepsy: from mechanisms to modeling

**DOI:** 10.3389/fneur.2026.1779207

**Published:** 2026-06-03

**Authors:** Kairan Zhao, Mengmeng Wu, Jun Zhao, Yiliang Teng, Zhen Sun, Yanping Sun

**Affiliations:** 1 Department of Neurology, The Affiliated Hospital of Qingdao University, Qingdao, China; 2 Department of Neurology, Qingdao Shinan Integrated Chinese and Western Medicine Hospital, Qingdao, China

**Keywords:** cross-frequency coupling, epilepsy, high-frequency oscillations, Neural Mass Models, sleep spindles

## Abstract

High-frequency oscillations (HFOs) and sleep spindles are key neural rhythms in non-rapid eye movement (NREM) sleep with significance for information processing and epilepsy research. This review summarizes the neurophysiological basis of HFOs and spindles, with emphasis on cross-frequency coupling and its pathological alterations in epilepsy. Cross-frequency coupling reflects spatiotemporal coordination within thalamocortical–hippocampal networks and may serve as an electrophysiological marker for distinguishing physiological from pathological activity and localizing epileptogenic zones. To bridge the gap between clinical observation and underlying mechanisms, we evaluate the potential of Neural Mass Models (NMMs) in elucidating the generation and abnormal coupling of these oscillations. Ultimately, the integration of multimodal electrophysiology and computational modeling offers a transformative pathway toward enhancing diagnostic precision and personalizing therapeutic interventions in epilepsy.

## 1 Introduction

Epilepsy is a chronic neurological disorder characterized by abnormal synchronous discharge of brain neurons, affecting about 50 million people worldwide and imposing a heavy burden on patients’ quality of life and society (1, 2). Current antiepileptic drugs and surgical interventions have achieved progress in seizure control. Approximately 30% of patients remain classified as drug-resistant epilepsy (DRE), and some present varying degrees of functional impairment, which highlights the limitations of existing treatments and their severe impact on long-term prognosis (3, 4). Therefore, precise localization of epileptogenic zones, which represent the volume of brain tissue responsible for generating seizures and requiring removal to achieve a seizure-free outcome, and elucidation of seizure mechanisms, particularly their impact on cognitive function, represent key research directions in neuroscience and clinical neurology.

High-frequency oscillations (HFOs) are brief EEG events with frequencies ranging from 80 to 600 Hz (5, 6). Initially identified in the hippocampus of animals (7, 8), research has since extended to human intracranial EEG (iEEG) and non-invasive EEG/MEG. HFOs occur frequently during non-rapid eye movement (NREM) sleep in healthy individuals, while fast ripples, defined as pathological oscillations typically ranging from 250 to 600 Hz, are more prevalent in epileptogenic regions, highlighting their critical role in epilepsy development. Sleep spindles are rhythmic 11–16 Hz bursts generated by thalamocortical circuits. As a hallmark sleep rhythm, they exhibit cross-frequency coupling (CFC) with HFOs—a phenomenon in which the phase or amplitude of one frequency band modulates another—to maintain neural network coordination. In epilepsy, this physiological coordination is frequently replaced by abnormal coupling between epileptiform high-frequency activities and sleep spindles, which can lead to widespread network dysfunction and is associated with potential cognitive impairment (9). Elucidating the mechanisms underlying HFOs, spindles, and their coupling is essential for understanding epileptogenic processes and advancing precision diagnosis and individualized treatment.

In recent years, computational modeling, particularly Neural Mass Models (NMMs), has emerged as a valuable theoretical framework for exploring cross-frequency coupling, which provide a phenomenological bridge to integrate multiscale findings, translating hypotheses regarding synaptic excitability into observable macroscopic clinical EEG patterns (10–13). This review first introduces the neurophysiological basis of HFOs and sleep spindles, highlighting how their hierarchical coupling orchestrates normal information processing. We then detail how this coupling is pathologically altered in epilepsy and review its clinical significance for epileptogenic zone localization. Following the review of neural physiology, Neural Mass Models (NMMs) and related computational approaches are introduced as low-dimensional dynamical systems that effectively balance physiological realism with computational tractability. Ultimately, we propose that these models serve as an essential translational bridge, offering mechanistic insights and technical support for precision medicine in epilepsy.

## 2 Neural mechanisms of HFOs

### 2.1 Definition and origins of HFOs

**Figure 1:**
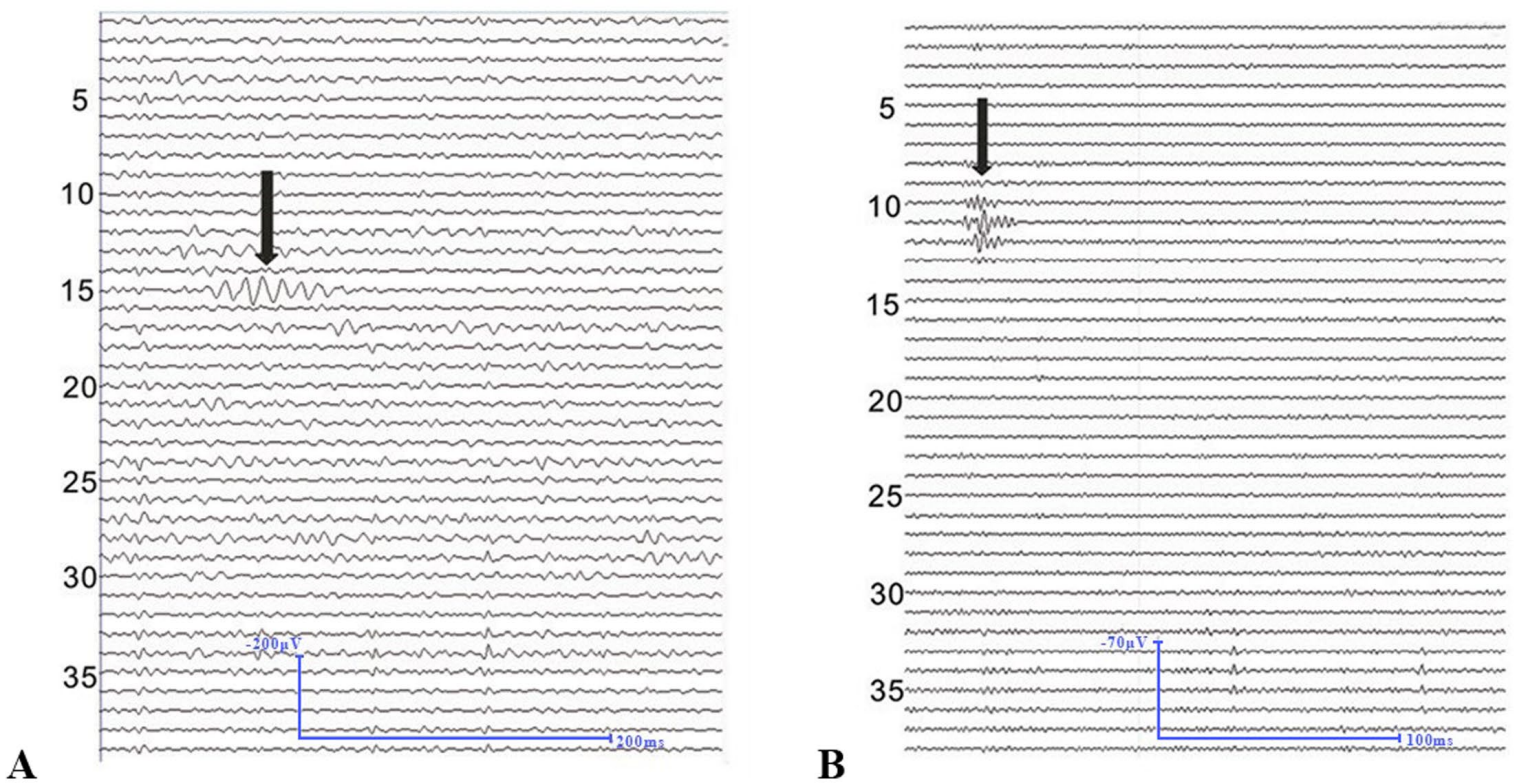
Ripple and fast ripple analyzed by Advanced Source Analysis. **(A)** A physiological ripple (typically 80–250 Hz), characterized by rhythmic oscillatory events. These are often observed in healthy hippocampal or neocortical networks and are associated with memory consolidation. **(B)** A pathological fast ripple (typically >250 Hz), manifesting as a disorganized, high-frequency burst. These events are specifically localized to the epileptogenic zones and serve as a biomarker for epileptic tissue. In both panels, the y-axis represents the voltage amplitude (in μV), with vertical scale bars indicating −200 μV for **(A)** and −70 μV for **(B)**. The horizontal scale bars represent 200 ms and 100 ms, respectively. Data were strictly preprocessed, and artifactual channels were excluded by visual inspection. To isolate these specific oscillations from the background activity, raw EEG signals were band-pass filtered (80–250 Hz for ripples; 250–500 Hz for fast ripples). All high-frequency oscillations were detected, analyzed, and visualized using Advanced Source Analysis (ASA) software. The arrows indicate the specific oscillatory events within the EEG traces (26). (Reproduced Citation: Sun et al., 2015; License: Creative Commons Attribution 4.0 International License (CC BY 4.0); Source: Chinese Medical Journal).

HFOs refer to electrical activity at frequencies above the conventional EEG range, typically exceeding 80 Hz. Based on frequency range and functional characteristics, they are further classified into two types: (1) Ripples: 80–200 Hz or 80–250 Hz, commonly observed in the hippocampus and neocortex of healthy individuals, considered crucial for memory consolidation and information processing during sleep—in animal models, ripple occurrence is often closely synchronized with spatial memory replay; (2) Fast ripples: 200–500 Hz or higher, frequently seen in patients with epilepsy, particularly localized in epileptogenic zones, often indicating potential abnormal network discharges. These classification criteria are widely applied in clinical and basic neuroscience, and different types of HFOs are believed to originate from distinct neural circuit mechanisms (14, 15) (Figure 1).

The neural origins of HFOs can be traced to the firing properties of individual neurons as well as to large-scale network dynamics. Under physiological conditions, ripples are typically driven by synchronous activity between inhibitory and excitatory neurons, particularly dependent on the interaction between pyramidal neurons and inhibitory interneurons within the cortico-hippocampal circuit (16). In pathological conditions, fast ripples often lose this synchrony, exhibiting asynchronous discharges in neuronal populations; in epileptogenic zones, such abnormalities usually appear as dense clusters of high-frequency discharges (17, 18), representing an important signal of local network pathology. HFOs generation relies on specific structural substrates, with the hippocampal CA1 and CA3 regions being hotspots for ripples, frequently observed during sleep and memory-related tasks (19). The thalamo-cortical pathway plays a key role in regulating the rhythmicity of cortical HFOs (20), indicating that HFOs are not purely local phenomena but are also influenced by long-range network dynamics. The primary sources of fast ripples are often localized to cortical epileptogenic zones, a feature confirmed in multiple clinical studies and recognized as an important biomarker for surgical localization in epilepsy (21–23).

### 2.2 Functions and pathological significance of HFOs

HFOs are not merely electrophysiological reflections of microscopic neural dynamics but are essential components of the brain’s information processing across different states. Based on their physiological properties and spatial distribution, HFOs can be broadly classified into physiological and pathological types, with functional differences closely related to their localization and regional distribution.

In healthy individuals, ripples are widely observed in the hippocampus, parietal cortex, and prefrontal cortex, playing important roles in cognitive processing and memory functions. During slow-wave sleep (SWS), ripples accompany the hippocampal-cortical replay process, regarded as a core neural mechanism of memory consolidation (24). Recent studies have shown that HFOs exhibit synchronous bursts across the frontal, temporal, and occipital lobes during memory retrieval tasks, indicating a critical role in cross-regional information integration (24, 25). This synchronous activity shows not only distinct spatial patterns but also precise temporal coordination, suggesting that long-range circuit integration may be mediated through relay structures such as the thalamus (24).

**Figure 2:**
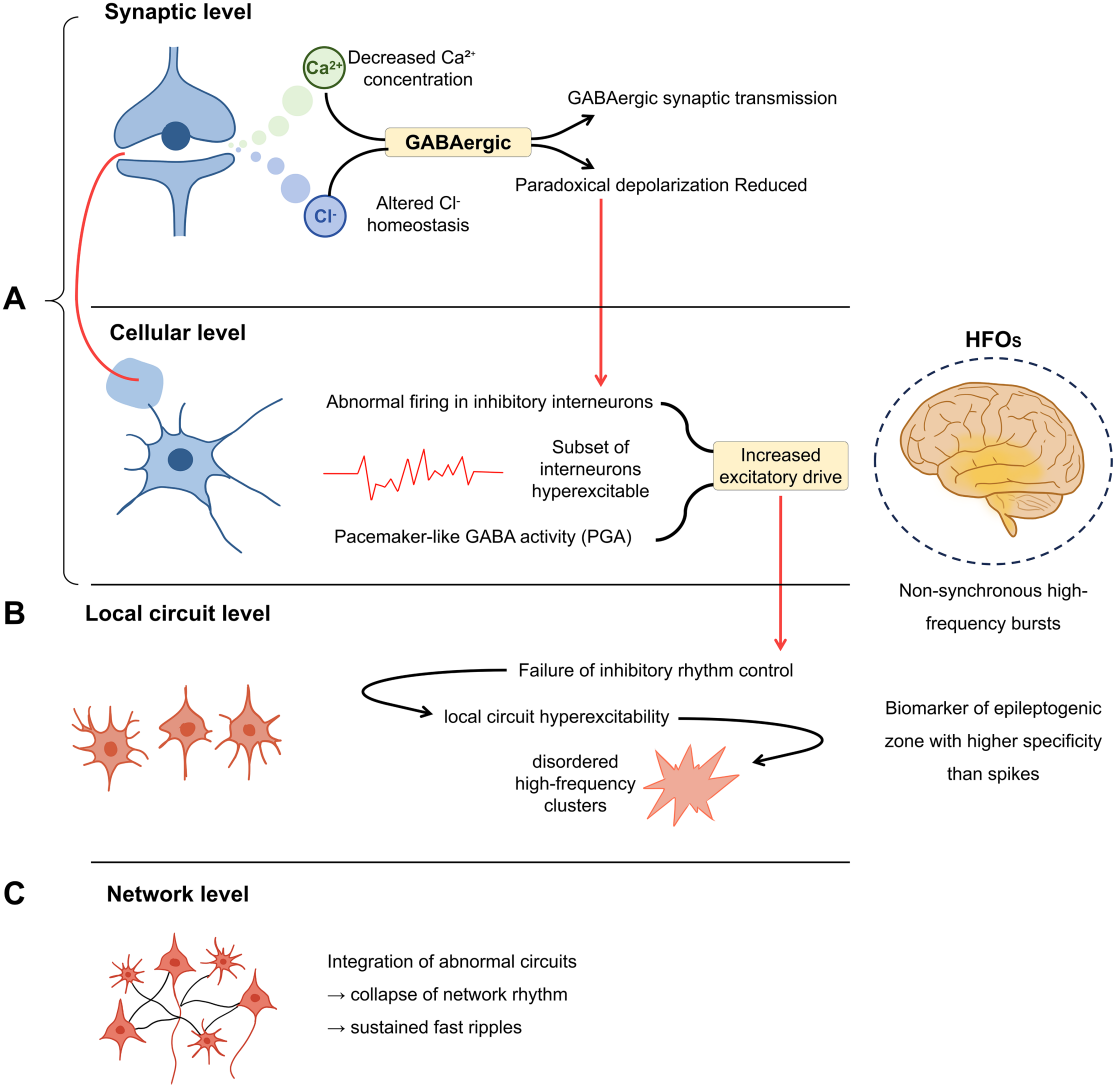
The figure illustrates the multi-scale progression of epileptogenic activity. **(A)** Microscopic and Synaptic Level: at the cellular scale, specific physiological alterations—such as decreased Ca^2+^ concentration and disrupted Cl^−^ homeostasis—lead to dysfunctional GABAergic synaptic transmission and paradoxical depolarization. These synaptic changes induce abnormal firing in inhibitory interneurons, characterized by Pacemaker-like GABA activity (PGA), which severely disrupts the local excitatory-inhibitory (E/I) balance. **(B)** Transition to Network Dynamics: These localized synaptic perturbations propagate across the mesoscopic neural mass, synchronizing local populations. **(C)** Macroscopic EEG Manifestations: Consequently, at the network level, this hyper-synchrony manifests as visible epileptiform spikes (interictal epileptiform discharges) and pathological cross-frequency coupling on the macroscopic EEG.

Unlike physiological HFOs, fast ripples typically occur in epileptogenic zones and manifest as asynchronous clusters of high-frequency discharges. This type of HFOs is considered an important biomarker of the epileptogenic zones (21, 26, 27). In presurgical evaluation for epilepsy, the spatial distribution of HFOs is often used to localize the seizure onset zone (SOZ). Early clinical studies have provided preliminary evidence that resection of the majority of HFO-generating regions—particularly pathological HFOs associated with interictal epileptiform discharges—may correlate more strongly with favorable post-surgical outcomes compared to the resection of spike-generating regions or the SOZ alone. This suggests that HFOs may offer additional spatial specificity in identifying epileptogenic tissue (22, 23, 28, 29). The underlying cause of pathological HFOs lies in the disruption of the excitation–inhibition balance within neural networks. Inhibitory interneurons in epileptogenic zones often exhibit abnormal firing patterns, breaking the original rhythmic control and triggering local high-frequency synchronous activity (15, 21). In healthy neural networks, the generation of high-frequency oscillations (such as physiological ripples) relies heavily on the precise, synchronized pacing provided by fast-spiking inhibitory interneurons and their rapid GABAergic signaling onto pyramidal cells. This delicate rhythmic inhibition strictly depends on maintaining a low intracellular chloride concentration to ensure that GABA receptor activation remains hyperpolarizing. However, in epileptogenic zones, this fundamental molecular architecture breaks down. Disruption of Cl^−^ homeostasis pathologically shifts the chloride reversal potential. Consequently, massive GABAergic inputs—such as those driving the inhibition-dominated UP-DOWN transition of slow waves—paradoxically induce excitatory depolarization rather than inhibition. This provides a potential mechanism for why pathological HFOs frequently occur during this phase (30, 31), although recent findings indicate that such phase preference is not exclusively pathological (32). Additionally, hyperexcitability of GABAergic interneurons and the presence of pacemaker-like GABA activity (PGA) provide a sustained drive for fast ripple generation. A reduction in GABAergic synaptic transmission due to decreased Ca^2+^ concentration, combined with enhanced glutamatergic synaptic transmission, can further promote pathological HFOs (33). Fast ripples may also arise from synchronous discharges of glutamatergic principal neurons (34). These changes contribute to the formation of pathological HFOs and represent network-level alterations that provide the biological framework for subsequent computational analysis. By abstracting these synaptic dynamics into tunable mathematical parameters, Neural Mass Models (detailed in Section 5) offer a way to quantitatively link empirical physiology with dynamic simulation (Figure 2).

## 3 Neurophysiological basis of sleep spindles

### 3.1 Definition and generation of sleep spindles

Sleep spindles appear on EEG as brief, high-amplitude oscillations at 11–16 Hz, lasting approximately 0.5–2 s, predominantly during NREM stage 2 (35). They are classified into slow spindles (11–13 Hz, frontal region) and fast spindles (13–16 Hz, parietal region) based on frequency and spatial distribution. Researchers quantify spindle characteristics using metrics such as density, mean duration, peak frequency, and instantaneous amplitude. Advanced analyses examine their phase relationships with slow waves or high-frequency oscillations (phase-locking index), as well as microstructural features like waveform shape and spectral energy (36–38).

Sleep spindle generation depends on the interaction between thalamocortical relay (TCR) neurons and the thalamic reticular nucleus (TRN), a specialized inhibitory GABAergic structure that envelops the thalamus (39). Inhibitory neurons in the TRN hyperpolarize TCR cells via GABA_A receptor-mediated phasic inhibition, activating low-threshold T-type calcium channels (Cav3.x) and triggering rebound burst firing (39), producing rhythmic 11–16 Hz oscillations. The corticothalamic circuit modulates thalamic rhythms through cortical projections, conferring local spindle generation and enabling widespread network synchronization. Notably, cortical projections can both enhance and selectively suppress spindle activity in specific frequency bands, providing rhythmic regulation across sleep stages and task demands (35). Additionally, distinct cell subtypes (e.g., PV + inhibitory neurons) and ion channels (T-type calcium, HCN channels) critically influence spindle frequency and duration (35).

### 3.2 Thalamocortical structural influence

The spatial distribution of sleep spindles is constrained by the thalamocortical circuit architecture rather than randomly dispersed across the cortex. Studies show that spindle power and density largely depend on the integrity of white matter microstructure, particularly fiber tracts constituting the thalamocortical pathway (40, 41). Functional MRI reveals that corticothalamic functional connectivity explains significant interindividual differences in spindle features such as density and peak frequency (42). In patients with focal epilepsy, spindle density is markedly reduced, with more pronounced decreases in regions associated with the epileptogenic zones. The effect is most significant on fast spindles (12–16 Hz) (43–45). Spindle generation primarily depends on synchronized oscillations within the thalamocortical circuit. Epilepsy-related network abnormalities may disrupt this dynamic balance, thereby limiting spindle generation and cross-regional propagation (46). Disruption of this circuit reflects not only local electrophysiological abnormalities but also impaired functional integration across brain regions, exacerbating deficits in cognitive processes such as memory processing and executive control (47). These alterations provide key physiological parameters, including thalamocortical connectivity strength and inhibitory function of the thalamic reticular nucleus, which can be directly incorporated into computational models to better simulate the dynamics of spindle activity under epileptic conditions.

## 4 Cross-frequency dynamics of HFOs and sleep spindle coupling

### 4.1 Physiological HFOs-spindle coupling

Our understanding of cross-frequency coupling between high-frequency oscillations (specifically physiological ripples) and sleep rhythms stems from foundational animal studies. Early *in vivo* recordings identified distinct high-frequency activities in the hippocampus, such as the initial observations of high-frequency network oscillations in cats (48) and the discovery of sharp-wave-associated ripples in the rat hippocampus (49, 50). Furthermore, hippocampal ripples orchestrate the reactivation of neuronal ensembles (replay) that were active during prior waking behavior, serving as a core mechanism for spatial memory consolidation (51). Building upon this framework, studies employing causal manipulations have provided converging evidence linking spindle-ripple coupling to behavioral outcomes. Latchoumane et al. (52) demonstrated that optogenetically inducing thalamic spindles phase-locked to slow oscillation up-states enhances their nesting of hippocampal ripples and promotes memory consolidation in mice. Complementing this, Geva-Sagiv et al. (53) showed that real-time closed-loop stimulation synchronized to medial temporal lobe slow waves in humans enhances spindle activity, improves ripple-thalamocortical coupling, and boosts recognition memory accuracy. Together with evidence from rodent timed stimulation studies (54), these findings confirm that precise temporal coordination of nested oscillations is essential for effective memory transfer. These pivotal animal models established that ripples (typically 140–200 Hz in rodents) naturally occur during slow-wave sleep (SWS) and immobility, representing highly synchronous population bursts. Human intracranial EEG (iEEG) has revealed that CFC between physiological ripples and sleep spindles is a central mechanism in memory research. Phase-amplitude coupling (PAC) analysis shows that ripples often occur near the trough (~180°) of spindles, coinciding with increased thalamocortical excitability that facilitates hippocampal-cortical information transfer (55). In healthy individuals, approximately 50–80% of detected hippocampal ripples synchronize with sleep spindles, with high-frequency activity amplitude increasing over 30% at spindle peak phases (55). Furthermore, hippocampal and cortical ripple bursts show temporal coupling with sleep spindles and slow-wave oscillations. This multilayered frequency integration is considered a critical physiological basis for memory trace transfer and consolidation (55–57).

**Figure 3:**
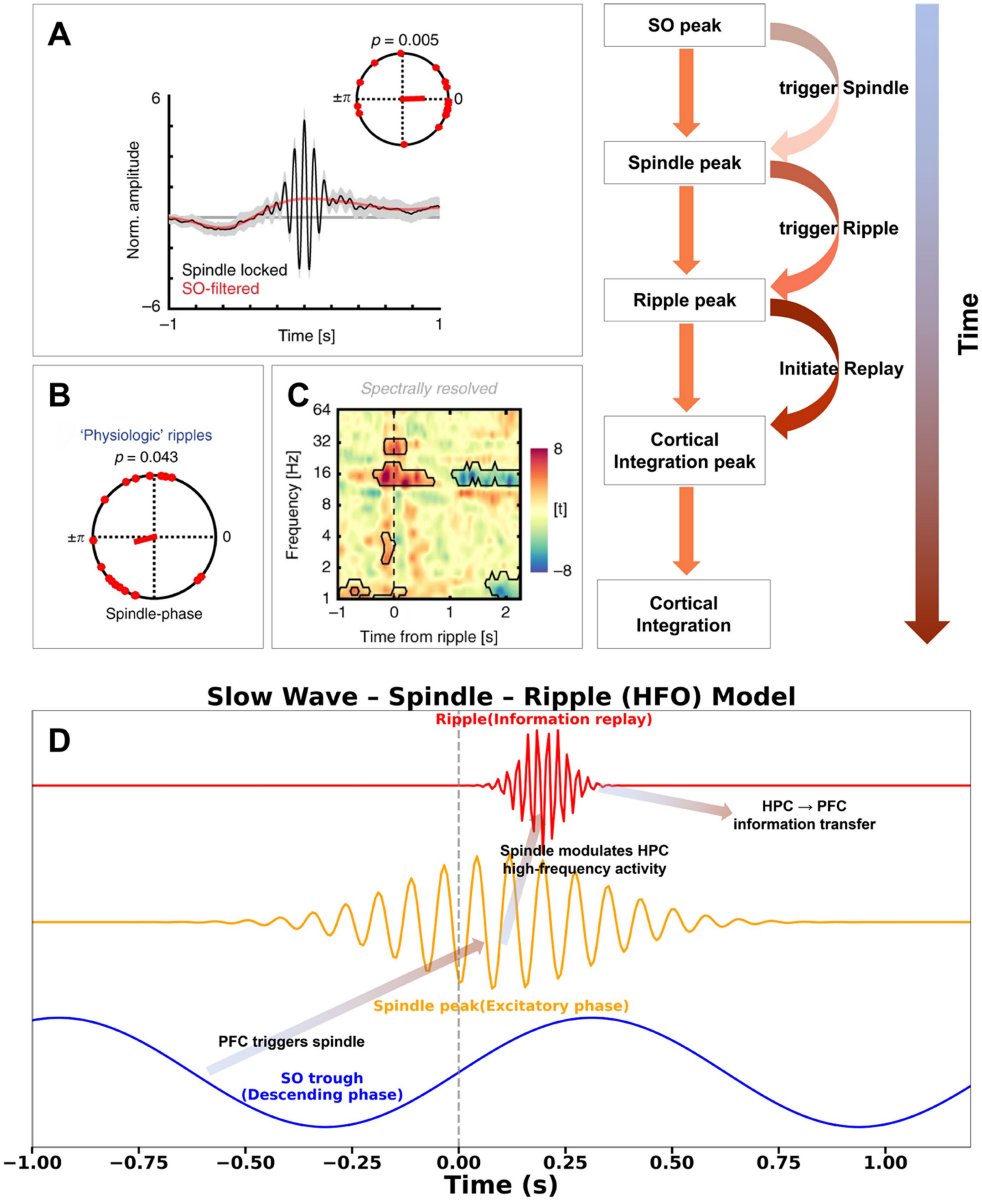
Sequential process of the SO-spindle-HFOs triple coupling model. **(A)** Sleep spindles are nested within the peak phase of slow oscillations (SO), reflecting thalamocortical activation during the high-excitability phase of SO (59); **(B)** Physiological hippocampal ripples cluster at the trough of spindles (~180°), when cortico-thalamo-hippocampal integration is maximal (59); **(C)** Approximately 1 s after ripples, hippocampal-to-cortical information flow increases significantly (59) (License: Creative Commons Attribution 4.0 International License (http://creativecommons.org/licenses/by/4.0/). The image has been cropped to fit the page layout). **(C)** Schematic of the triple coupling process.

The “slow oscillation-spindle-HFOs” triple coupling model reveals a nested temporal sequence: the slow wave’s down phase triggers spindles, whose excitatory peak induces hippocampal ripples, facilitating information replay and integration from the hippocampus to the cortex (58, 59) (Figure 3). This sequence recurs during slow-wave sleep (SWS) and is regarded as a key neural mechanism of long-term memory consolidation.

Regarding the electrophysiological evidence for this model, spatially, cortical regions contribute unevenly to the triple coupling (60). Both frontal and parietal spindles synchronize with hippocampal ripples, with stronger coupling between parietal spindles and hippocampal ripples. Frontal EEG shows significantly higher spindle density than parietal EEG (2.7 ± 0.1 vs. 2.0 ± 0.1 events/min), indicating functional heterogeneity in the thalamocortical-hippocampal network (61). Notably, Jacobs et al. (14) emphasized that physiological ripples are not strictly confined to the hippocampus but are also distributed across various neocortical areas. This widespread neocortical presence indicates that physiological HFOs are fundamental components of broader brain activity, laying the groundwork for further investigations into how these distributed fast oscillations integrate with macroscopic sleep rhythms (14).

### 4.2 Abnormal coupling in pathological conditions

Recent studies have profoundly refined our understanding of how high-frequency activities relate to cortical slow waves (SO). Under physiological conditions, normal ripples preferentially occur during the DOWN-UP transition (the onset of the active UP state) of the slow wave, seamlessly integrating into the classic slow wave-spindle-ripple tripartite organization (55). Conversely, pathological fast ripples exhibit a markedly divergent phase preference. As highlighted by Frauscher et al., Weiss et al., and Song et al., pathological ripples are significantly more likely to occur during the UP-DOWN transition—a period characteristically dominated by increasing cortical inhibition (21, 62, 63). Crucially, Song et al. established that when pathological ripples occur at this UP-DOWN transition, they fail to exhibit the physiological tripartite coupling with slow waves and spindles, thereby fundamentally disrupting sleep-dependent network communication.

In epilepsy and other neurological disorders, pathological fast ripples (typically >200 Hz) and interictal epileptiform discharges (IEDs) significantly disrupt normal cross-frequency coupling with sleep spindles. Pathological high-frequency oscillations (HFOs), particularly fast ripples, have been shown to be closely associated with the SOZ and epileptogenic zones, with their generation reflecting abnormal synchronous burst firing of principal neurons within epileptogenic tissue (14). Further mechanistic investigations have demonstrated that fast ripples (250–600 Hz) are preferentially localized to brain regions capable of generating spontaneous seizures—including the dentate gyrus, hippocampus, and entorhinal cortex—and that their occurrence is largely independent of structural lesions but strongly linked to functional epileptogenicity (15). However, the impact of the epileptic network on spindle generation is highly complex and dynamic. Several studies document a marked reduction in spindle density within epileptogenic regions (43, 64). Yet, the specific interaction between interictal epileptiform discharges (spikes) and spindles is decidedly bidirectional. While some research demonstrates that spikes can disrupt and truncate ongoing sleep spindles (45), other findings indicate that they can paradoxically induce spindle activity (65, 66). This heterogeneity suggests that whether the epileptogenic network suppresses physiological rhythms or aberrantly recruits them heavily depends on the specific state and structural integrity of the affected thalamocortical networks. Epileptic foci show an increased occurrence of pathological fast ripples accompanied by abnormally enhanced phase-amplitude coupling (PAC), such as aberrant *θ*/*α* − *γ* coupling. This abnormal PAC exhibits a distinct spatial gradient. It intensifies closer to the epileptogenic tissue (defined as the extended neural tissue capable of generating epileptic seizures) and peaks within the epileptogenic zones (63, 67). In frontal lobe epilepsy, high-PAC channels during interictal and preictal periods are predominantly distributed within the SOZ and its adjacent regions (67). In mesial temporal lobe epilepsy, PAC between epileptiform spike phase and pathological ripple amplitude typically demonstrates greater phase-locking strength and higher occurrence probability within the SOZ compared with non-SOZ areas (63). However, this spatial distribution pattern shows a certain degree of heterogeneity across patients and analytical approaches. Overall, the spatial clustering of abnormal PAC may reflect a key electrophysiological property of focal epileptic networks, although its stability and generalizability require further systematic validation.

The transition from physiological to pathological coupling can be viewed as a “mechanistic hijacking” of endogenous coordination frameworks (68). Instead of physiological ripples accurately locking to the optimal excitatory phase (the trough) of sleep spindles to facilitate memory transfer, pathological fast ripples exhibit phase-shifts. They override the inhibitory gating typically provided by the thalamocortical circuits, discharging asynchronously or coupling with the wrong phase of the slow oscillation. In temporal lobe epilepsy models, hippocampal interictal epileptiform discharges (IEDs) exhibit abnormal synchronization with spindles, replacing normal ripple-spindle coupling and resulting in impaired memory consolidation (69–71).

Physiological ripples and pathological fast ripples significantly overlap within the 200–250 Hz frequency range, making frequency alone insufficient for accurate differentiation. Identification requires integrating cross-frequency coupling features, spatial distribution, and sleep stage information (72, 73). Noninvasive EEG may underestimate cortical fast ripple-spindle coupling strength above 100 Hz due to low signal-to-noise ratio (74). Furthermore, while physiological ripple-spindle coupling is well recognized as a critical neural mechanism for sleep-dependent memory consolidation (75), the specific cognitive impact of its pathological counterpart in human epilepsy remains under exploration. Existing epilepsy research has primarily focused on validating pathological high-frequency oscillations (HFOs), particularly fast ripples, as biomarkers of epileptogenicity and seizure severity (14, 15). Although these studies confirm a robust spatial correlation between abnormal discharges and the SOZ, demonstrating clinical value for surgical prognosis, extrapolating directly from this spatial association to establish a causal link with cognitive impairment overlooks the complex intermediate processes between spatial distribution and functional disruption. Consequently, how abnormal coupling patterns interfere with network information flow to induce cognitive deficits awaits direct empirical validation. Future research requires longitudinal, multicenter, and multimodal designs (e.g., SEEG, MEG, fMRI combined with behavioral assessment) integrated with computational modeling to mechanistically test the emergence of abnormal coupling patterns and their disruption of network communication.

### 4.3 Clinical value of cross-frequency coupling

Fast ripples have emerged as important electrophysiological markers for presurgical localization of epileptogenic zones (21). Incorporating spindle coupling characteristics holds significant potential to refine the accuracy of epileptogenic zone identification. The strength and spatial distribution of abnormal fast ripple-spindle coupling may also guide surgical resection boundaries and inform postoperative prognosis (21, 22). For instance, Cai et al. (76) identified that pathological HFOs stably co-occurring with spikes are highly specific to the epileptogenic zones, significantly improving source imaging localization accuracy and correlating with postoperative recurrence risk. Similarly, Liu et al. (77) proposed a multi-dimensional feature fusion system combining sLORETA and deep learning, providing a scalable screening tool for drug-resistant epilepsy. Regarding cognitive outcomes, emerging evidence underscores the critical role of thalamocortical spindle dynamics. In a compelling study on sleep-activated developmental epilepsy, Kramer et al. (47) demonstrated that focal sleep spindle deficits serve as a robust predictor of cognitive impairment, highlighting the vulnerability of these networks. Building on such network dysfunction, Gelinas et al. (69) found that interictal epileptiform discharges (IEDs) can disrupt the precise coordination between ripples and spindles, effectively ‘coupling’ with spindles in a way that impairs memory consolidation. Recently, Yu et al. (9) recently observed that abnormal coupling between pathological fast ripples and sleep spindles extends beyond the epileptogenic zones, correlating with both cognitive decline and poor seizure control in pediatric focal epilepsy. However, consistent with the need for rigorous mechanistic validation, large-scale longitudinal studies are still required to firmly establish this coupling pattern as a definitive early neurophysiological biomarker for cognitive decline, rather than just a spatial correlate (Table 1).

**Table 1:** Comparison of physiological and pathological high-frequency oscillation (HFO) coupling with sleep spindles.

Feature	Physiological ripple-spindle coupling	Abnormal spindle-HFO coupling
Frequency range	Ripples: typically 80–200 Hz (centered ~100–150 Hz in humans).	Pathological Ripples (80–250 Hz) and Fast Ripples (250–500 Hz).
Spindle characteristics	Globally coordinated, typical duration/amplitude; facilitates widespread cortical network communication.	Often spatially disrupted or functionally altered; local spindle deficits may occur in some epilepsies.
Coupling phase	Highly precise temporal nesting; ripples are phase-locked to the troughs of spindles.	Disrupted phase preference; loose coupling or “hijacking” of the spindle phase by epileptiform discharges.
Animal/human evidence	Animals: extensive optogenetic/iEEG data.	Animals: seizure models (e.g., kindling, pilocarpine).
	Humans: robust iEEG/scalp EEG evidence during sleep memory tasks.	Humans: extensive iEEG evidence linking pathological HFOs to the SOZ.
Behavioral correlation	Positively correlates with sleep-dependent memory consolidation and cognitive performance.	Correlates with seizure severity, epileptogenicity, and cognitive decline/memory deficits.
Causal validation	Established. Closed-loop disruption of the ripple-spindle sequence actively impairs memory consolidation (e.g., in rodents).	Emerging hypothesis. Mechanistic causality for cognitive impairment remains largely theoretical in humans, awaiting direct empirical and computational validation.

While physiological coupling is a well-established mechanism for memory consolidation with robust causal evidence in animal models, the specific cognitive consequences of pathological fast ripple-spindle coupling in humans currently represent an emerging hypothesis requiring further longitudinal and causal validation. HFO, High-frequency oscillation; SOZ, Seizure onset zone; iEEG, intracranial electroencephalography.

## 5 Clinical and computational modeling applications

### 5.1 Rationale for computational modeling

Despite detailed observations of the “slow oscillation-spindle-ripple” sequence, empirical iEEG recordings cannot directly disentangle the underlying synaptic kinetics—specifically, the precise balance between excitatory pyramidal drive and inhibitory GABAergic pacing. To bridge this gap, computational frameworks such as NMMs are essential. By translating dynamic, multilayered physiological couplings into tunable mathematical parameters, NMMs offer a phenomenological testing ground grounded in mean-field dynamics for investigating both normal memory consolidation and its pathological breakdown. It should be emphasized that these parameters do not directly represent microscopic variables, but rather serve as effective mesoscopic approximations constrained by underlying cellular mechanisms.

Crucially, this shift from functional nesting to seizure-prone pathological synergy provides a theoretical basis for NMMs. While NMMs cannot directly test microscopic synaptic events, they utilize effective proxies—such as decreasing inhibitory synaptic gains (representing compromised GABAergic interneuron function) or altering synaptic time constants within the simulated thalamic reticular nucleus (TRN)—to simulate these conditions. Through this phenomenological approach, the “hijacking” of the spindle rhythm by fast ripples is investigated as a modelable dynamical transition. By tuning specific mathematical parameters—such as decreasing the inhibitory synaptic gains (*A*) or altering the synaptic time constants (*a*) within the TRN (see the relevant equations in Section 5.2)—researchers can evaluate whether specific population-level shifts are sufficient to reproduce these pathological phase-shifts in silico. Thus, NMMs serve as an essential bridge, allowing us to test how altering effective mesoscopic proxies (reflecting underlying microscopic synaptic failures) leads to macroscopic decoupling of the slow wave-spindle-fast ripple sequence.

### 5.2 Formulating the NMM for cross-frequency coupling

Computational modeling of epilepsy spans multiple scales, ranging from detailed biophysical simulations of single neurons to macroscopic mean-field approximations. Detailed biophysical models, such as those based on the Hodgkin-Huxley formalism, and Spiking Neural Networks (SNNs) offer superior granularity by simulating ion channel dynamics and precise spike timing at the single-neuron level (78, 79). These micro-scale models are invaluable for investigating cellular mechanisms, such as the specific impact of channelopathies on neuronal excitability. However, their high computational cost and the “parameter explosion” problem—where thousands of unobservable parameters must be estimated—make them challenging to scale for whole-brain simulations or clinical applications (80). In contrast, NMMs reduce this complexity by modeling the mean-field dynamics (average firing rates and membrane potentials) of neuronal populations rather than individual cells. This simplification allows for a direct mapping to macroscopic electrophysiological recordings (EEG/SEEG) and facilitates the efficient inference and fitting of model parameters from patient data, representing a strategic balance between biological realism and computational tractability for epilepsy research (81). Given these advantages, NMMs—rooted in the foundational works of Wilson and Cowan (82), and later refined by Jansen and Rit (83), have become the primary framework for investigating cross-frequency phenomena in this field.

NMMs can be broadly described as low-dimensional dynamical systems. While modern literature offers diverse mathematical formulations to capture specific physiological details (84), classical frameworks (such as the foundational Jansen-Rit model) typically rely on two fundamental operations represented by coupled ordinary differential equations (ODEs), which model the continuous-time evolution of neuronal state variables (83).

First, a linear synaptic filter converts the average presynaptic firing rate m(t) into an average postsynaptic membrane potential V(t) using a second-order ODE:


$$\frac{d^{2}V(t)}{dt^{2}}+2a\frac{\mathrm{dV}(t)}{\mathrm{dt}}+a^{2}V(t)=A\cdot a\cdot m(t)$$

Here, A represents the maximum synaptic gain (excitatory or inhibitory amplitude), and a is the inverse of the synaptic time constant ( τ=1/a ).

**Figure 4:**
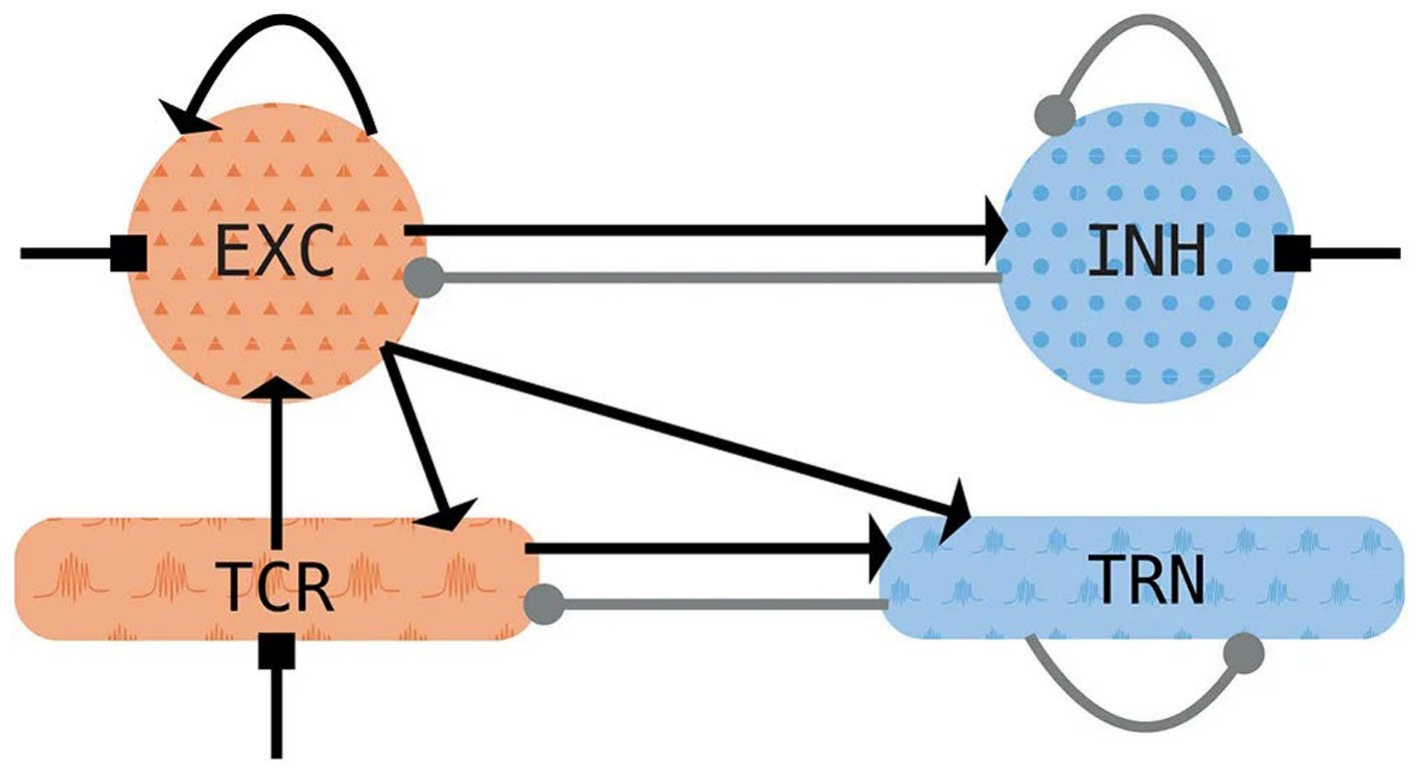
Schematic representation of a biophysically realistic thalamocortical Neural Mass Model (NMM). The model simulates the mean-field dynamics of four interacting neural populations: cortical excitatory pyramidal neurons (EXC), cortical inhibitory interneurons (INH), thalamocortical relay neurons (TCR), and the thalamic reticular nucleus (TRN). Black arrows indicate excitatory glutamatergic projections, which drive network activity and long-range cortico-thalamic feedback. Gray lines ending in circles represent inhibitory GABAergic projections, which are crucial for pacing sleep spindles (e.g., the TRN ⊣ TCR projection). In epilepsy research, tuning the mathematical parameters of these specific connections (such as decreasing the inhibitory synaptic gain of the TRN) allows for the mechanistic simulation of pathological fast ripple generation and the “hijacking” of physiological spindle coupling. (Reproduced from (86) under the Creative Commons Attribution 4.0 International License).

Second, a non-linear sigmoid function maps the mean membrane potential V(t) of the neural population back to an average output firing rate Q(t) :


$$Q(t)=\frac{Q_{\max}}{1+\exp[-r(V(t)-V_{\mathrm{th}})]}\;$$

where $Q_{\max}$ is the maximum firing rate, r is the steepness of the sigmoid curve, and $V_{\mathrm{th}}$ is the firing threshold. By dynamically tuning parameters such as the inhibitory gain ( $A_{\mathrm{inh}}$ ) or time constant ( $a_{\mathrm{inh}}$ ) within the simulated thalamic reticular nucleus (TRN), researchers can systematically model the mechanistic breakdown of spindle-ripple coupling during epileptic discharges (85, 86) (Figure 4).

**Figure 5:**
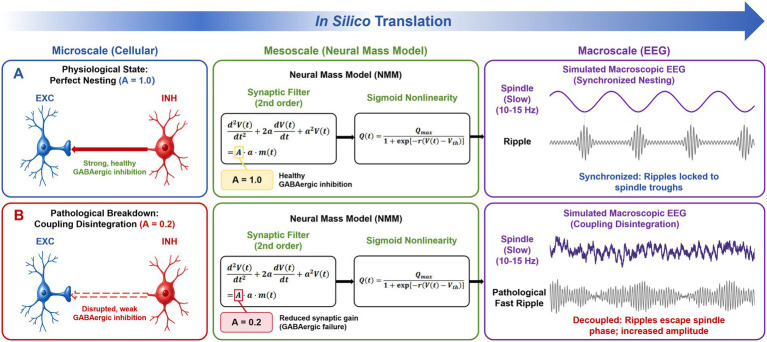
Model schematic: mapping microscopic synaptic gain to macroscopic coupling breakdown. **(A)** Physiological State: In healthy networks, the interaction between excitatory (EXC) and inhibitory (INH) populations is mathematically captured by a second-order synaptic filter [governing the mean membrane potential, V(t) ] and a nonlinear sigmoid function [converting potential to average firing rate, Q(t) ]. When the inhibitory synaptic gain parameter (*A*) is set to a robust physiological baseline (e.g., modeling intact GABAergic transmission), the simulated macroscopic EEG exhibits precise phase-amplitude nesting, with fast ripples tightly phase-locked to spindle troughs. **(B)** Pathological Breakdown: The microscopic synaptic failure is emulated by explicitly reducing the gain parameter (*A*) in the synaptic filter equation. This single population-level shift alters the E/I balance, propagating through the sigmoid transformation Q(t) . Consequently, the model predicts a macroscopic decoupling: fast ripples “escape” the spindle phase constraint, increasing in amplitude and disrupting the physiological sequence. This in silico mechanism directly links the mathematical parameters of the synaptic filter to the clinical observation of pathological synergy.

NMMs are particularly suited for this field because they can effectively represent interactions within the cortico-thalamo-hippocampal network (Figure 4), including the thalamic reticular nucleus (TRN), thalamocortical relay neurons (TCR), and cortical pyramidal neurons. Crucially, the mathematical parameters in these models correspond directly to mechanistic physiological variables. For instance, excitatory and inhibitory synaptic gains dictate the amplitude of population responses, while synaptic time constants determine the decay of postsynaptic potentials. By systematically tuning these parameters—such as reducing the inhibitory gain of the simulated TRN to mimic GABAergic impairment—researchers can mechanically test how altering these effective mesoscopic proxies generates macroscopic pathological high-frequency bursts and phase-shifts in spindle coupling (Figure 5).

To translate the aforementioned theoretical framework into computational practice, models must be carefully designed to capture the diverse timescales inherent in cross-frequency coupling. Recent literature reflects a progressive evolution in this domain, moving from foundational models of healthy physiological pacemakers to increasingly complex clinical applications. As a crucial first step in modeling these hierarchical interactions, Soplata et al. (87) demonstrated how detailed modeling of thalamocortical loops using Hodgkin-Huxley type neurons gives rise to phase-amplitude coupling, providing a computational template for how spindle phases dynamically gate higher-frequency bursts. Similarly, Tripathi and Gluckman (88) developed mechanistic Neural Mass (mNM) models by combining Hodgkin-Huxley type neurons with NMMs to robustly link cellular physiology to mean-field dynamics, proving that microscopic ion channel kinetics—which are crucial for understanding fast ripple generation—can be effectively integrated into macroscopic equations. Transitioning to clinical scenarios, studies have utilized NMMs to capture spindle and slow wave dynamics and their phase dependencies. Cona et al. (85) and Jajcay et al. (86) successfully simulated these interactions. Li et al. (89) proposed a Rolandic epilepsy NMM (RE-NMM), demonstrating that reductions in NMDA or h-type currents can trigger and promote epileptiform spike generation, while confirming the regulatory role of inhibitory output from the thalamic reticular nucleus (TRN) on spikes and spindles. Furthermore, Pan et al. (90) developed a pediatric focal epilepsy model (CFE-TCM) based on an improved Costa model, reproducing NREM spike-spindle coupling and showed this coupling is modulated by thalamocortical long-range connectivity and correlates with cognitive impairment. Building upon these successes in modeling low- and intermediate-frequency rhythms, the next critical step in computational epileptology is to incorporate the ultrafast synaptic kinetics required to explicitly simulate fast ripple-spindle coupling. Consequently, existing NMM frameworks provide a robust mathematical foundation for eventually achieving a computational theory of multi-band pathological interactions.

### 5.3 Multimodal integration and future prospects

**Figure 6:**
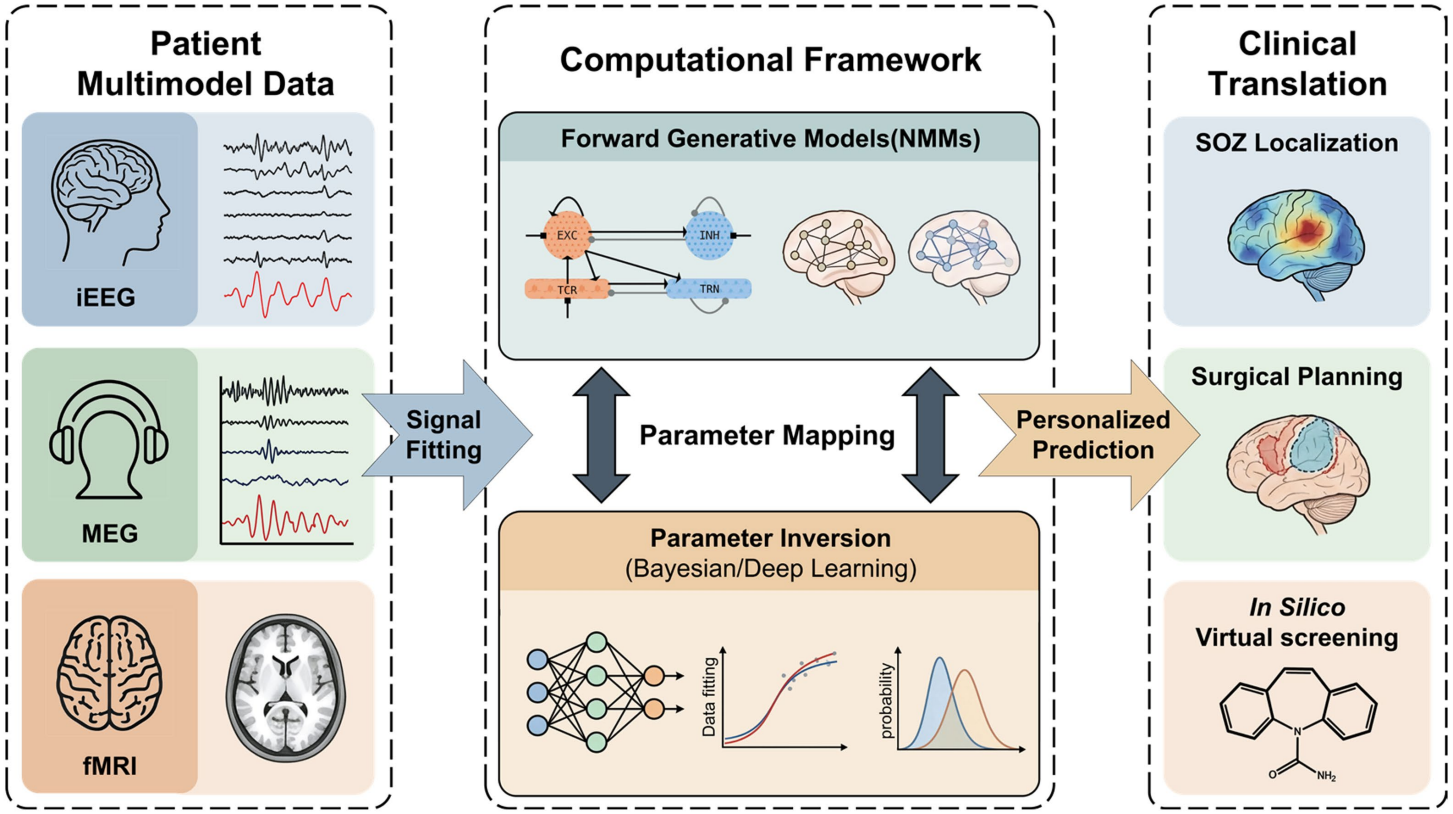
Schematic flowchart of the layered modeling framework. The workflow illustrates the integration of patient-specific multimodal data (iEEG, MEG, fMRI) into Neural Mass Models (NMMs). By solving the inverse problem through traditional Bayesian methods (e.g., DCM) or accelerated Deep Learning (DL) architectures, the framework maps macroscopic electrophysiological signals back to microscopic synaptic parameters. Ultimately, this in silico modeling supports clinical translation by providing quantitative guidance for SOZ localization, surgical boundary prediction, and the simulation of targeted neuromodulatory interventions.

A significant advantage of NMMs is their ability to serve as layered generative forward models, mapping hidden neural source activity to sensor-level signals like intracranial EEG (iEEG), MEG, and fMRI. Combined with Dynamic Causal Modeling (DCM) and Bayesian inversion methods (91, 92), NMMs allow for the estimation of individualized synaptic parameters based on a specific patient’s neuroimaging data. However, successful personalization relies heavily on the careful specification of model parameters that must be defined *a priori*. A significant challenge in this computational approach is that we often lack direct empirical evidence—such as human intracranial measurements or cellular-level recordings—to robustly justify the specific values assigned to these pre-set parameters. With such care taken, this personalized modeling holds the theoretical potential to predict coupling alterations under different surgical resection strategies, offering quantitative guidance for localizing the epileptogenic zones and balancing seizure control with functional preservation (Figure 6).

Moreover, accurately capturing a patient’s specific cross-frequency coupling dynamics paves the way for in silico therapeutic testing. Because the mathematical parameters of NMMs reflect underlying synaptic kinetics, these models provide a theoretical framework to simulate how targeted interventions—such as pharmacological agents or closed-loop neuromodulation—might effectively decouple fast ripples from sleep spindles and restore physiological oscillatory nesting (93, 94). This capability naturally extends the utility of NMMs from static presurgical localization to the dynamic optimization of patient-specific treatments.

While traditional Bayesian inverse methods are powerful for personalizing these models, they become computationally prohibitive when applied to advanced frameworks that incorporate detailed microscopic kinetics (88). To overcome this computational bottleneck and efficiently estimate these deeper microscopic parameters, recent integration with Deep Learning (DL) offers a powerful solution (95). By significantly accelerating the inverse problem, hybrid NMM-DL frameworks make large-scale, patient-specific parameter inference computationally feasible. While substantial work is needed to validate these models in clinical cohorts, accelerating these computational pipelines is a crucial step toward the long-term goal of personalized presurgical planning and the optimization of patient-specific interventions.

## 6 Summary

This review synthesizes current evidence demonstrating that the transition from physiological sleep rhythms to epileptic networks is marked by the pathological decoupling of the slow wave-spindle-HFOs sequence. While clinical iEEG and multimodal imaging have successfully established fast ripples and their abnormal coupling as biomarkers for the epileptogenic zone, empirical observation alone remains insufficient to unravel the underlying synaptic failures.

Here, we argue that computational modeling—specifically NMMs—is not merely an optional analytical tool, but an indispensable mechanistic framework. By conceptually linking microscopic synaptic alterations (such as impaired GABAergic inhibition or chloride dysregulation) to effective mesoscopic parameters that drive macroscopic mean-field dynamics, NMMs offer a promising theoretical framework to explore the gap between cellular pathology and macroscopic clinical EEG. This computational perspective allows the “hijacking” of spindle rhythms to be investigated beyond purely descriptive observations, providing a solid mathematical basis for future mechanistic testing.

Moving forward, the field must transition from descriptive correlation to predictive personalized modeling. Future research should prioritize longitudinal and multimodal experimental designs (e.g., integrating SEEG with MEG or fMRI) combined with hybrid NMM-Deep Learning frameworks. This layered modeling approach has the potential to mitigate the computational bottlenecks of traditional Bayesian inversion, facilitating more efficient parameter inference in clinical settings. Ultimately, such integration may help transition the utility of cross-frequency coupling from a static diagnostic biomarker toward a dynamic, computational target, potentially guiding personalized surgical boundaries and optimizing closed-loop neuromodulatory interventions.

## References

[1] Thurman DJ Logroscino G Beghi E Hauser WA Hesdorffer DC Newton CR . The burden of premature mortality of epilepsy in high-income countries: a systematic review from the mortality task force of the international league against epilepsy. Epilepsia. (2017) 58:17–26. doi: 10.1111/epi.13604, 27888514 PMC7004822

[2] World Health Organization. Epilepsy (2024). Available online at: https://www.who.int/news-room/fact-sheets/detail/epilepsy (accessed May 27, 2026)

[3] Holmes GL. Cognitive impairment in epilepsy: the role of network abnormalities. Epileptic Disord. (2015) 17:101–16. doi: 10.1684/epd.2015.0739, 25905906 PMC5410366

[4] Kwan P Brodie MJ. Early identification of refractory epilepsy. N Engl J Med. (2000) 342:314–9. doi: 10.1056/NEJM200002033420503, 10660394

[5] Buzsáki G Silva FLD. High frequency oscillations in the intact brain. Prog Neurobiol. (2012) 98:241–9. doi: 10.1016/j.pneurobio.2012.02.004, 22449727 PMC4895831

[6] Sun Y-P Liu Y Liu M Li S Wang Y. High-frequency oscillations in electroencephalography and epilepsy. Chin J Neurol. (2014) 47:421–3. doi: 10.3760/cma.j.issn.1006-7876.2014.06.015

[7] Bragin A Engel J Wilson CL Fried I Mathern GW. Hippocampal and entorhinal cortex high-frequency oscillations (100–500 Hz) in human epileptic brain and in Kainic acid-treated rats with chronic seizures. Epilepsia. (1999) 40:127–37. doi: 10.1111/j.1528-1157.1999.tb02065.x, 9952257

[8] Huang C White LE Jr. High-frequency components in epileptiform EEG. J Neurosci Methods. (1989) 30:197–201. doi: 10.1016/0165-0270(89)90130-1, 2607781

[9] Yu H Kim W Park DK Phi JH Lim BC Chae J . Interaction of interictal epileptiform activity with sleep spindles is associated with cognitive deficits and adverse surgical outcome in pediatric focal epilepsy. Epilepsia. (2024) 65:190–203. doi: 10.1111/epi.17810, 37983643 PMC10873110

[10] Chehelcheraghi M van Leeuwen C Steur E Nakatani C. A neural mass model of cross frequency coupling. PLoS One. (2017) 12:e0173776. doi: 10.1371/journal.pone.0173776, 28380064 PMC5381784

[11] Hyafil A Giraud A-L Fontolan L Gutkin B. Neural cross-frequency coupling: connecting architectures, mechanisms, and functions. Trends Neurosci. (2015) 38:725–40. doi: 10.1016/j.tins.2015.09.001, 26549886

[12] Liu Z Han F Wang Q. A review of computational models for gamma oscillation dynamics: from spiking neurons to neural masses. Nonlinear Dyn. (2022) 108:1849–66. doi: 10.1007/s11071-022-07298-6

[13] Tenney JR Williamson BJ Kadis DS. Cross-frequency coupling in childhood absence epilepsy. Brain Connect. (2022) 12:489–96. doi: 10.1089/brain.2021.0119, 34405685

[14] Jacobs J Staba R Asano E Otsubo H Wu JY Zijlmans M . High-frequency oscillations (HFOs) in clinical epilepsy. Prog Neurobiol. (2012) 98:302–15. doi: 10.1016/j.pneurobio.2012.03.001, 22480752 PMC3674884

[15] Staba RJ Bragin A. High-frequency oscillations and other electrophysiological biomarkers of epilepsy: underlying mechanisms. Biomark Med. (2011) 5:545–56. doi: 10.2217/bmm.11.72, 22003903 PMC3233380

[16] Jiruska P Alvarado-Rojas C Schevon CA Staba R Stacey W Wendling F . Update on the mechanisms and roles of high-frequency oscillations in seizures and epileptic disorders. Epilepsia. (2017) 58:1330–9. doi: 10.1111/epi.13830, 28681378 PMC5554080

[17] Engel J Jr Bragin A Staba R Mody I. High-frequency oscillations: what is normal and what is not? Epilepsia. (2009) 50:598–604. doi: 10.1111/j.1528-1167.2008.01917.x, 19055491

[18] Shamas M Benquet P Merlet I Khalil M El Falou W Nica A . On the origin of epileptic high frequency oscillations observed on clinical electrodes. Clin Neurophysiol. (2018) 129:829–41. doi: 10.1016/j.clinph.2018.01.062, 29482079

[19] Bagshaw AP Jacobs J LeVan P Dubeau F Gotman J. Effect of sleep stage on interictal high-frequency oscillations recorded from depth macroelectrodes in patients with focal epilepsy. Epilepsia. (2009) 50:617–28. doi: 10.1111/j.1528-1167.2008.01784.x, 18801037 PMC3792080

[20] Timofeev I Bazhenov M Seigneur J Sejnowski T. "Neuronal synchronization and thalamocortical rhythms in sleep, wake and epilepsy". In: Jaspers Basic Mech. Epilepsies Internet, 4th Edn Bethesda (MD): National Center for Biotechnology Information (US). (2012) 22787672

[21] Frauscher B Bartolomei F Kobayashi K Cimbalnik J Van ‘T Klooster MA Rampp S . High-frequency oscillations: the state of clinical research. Epilepsia. (2017) 58:1316–29. doi: 10.1111/epi.13829, 28666056 PMC5806699

[22] Schönberger J Frauscher B von Ellenrieder N Avoli M Dubeau F Gotman J. Fast ripple analysis in human mesial temporal lobe epilepsy suggests two different seizure-generating mechanisms. Neurobiol Dis. (2019) 127:374–81. doi: 10.1016/j.nbd.2019.03.030, 30928645

[23] Thomschewski A Hincapié A-S Frauscher B. Localization of the epileptogenic zone using high frequency oscillations. Front Neurol. (2019) 10:94. doi: 10.3389/fneur.2019.00094, 30804887 PMC6378911

[24] Kucewicz MT Cimbalnik J Garcia-Salinas JS Brazdil M Worrell GA. High frequency oscillations in human memory and cognition: a neurophysiological substrate of engrams? Brain. (2024) 147:2966–82. doi: 10.1093/brain/awae159, 38743818 PMC11370809

[25] Vaz AP Inati SK Brunel N Zaghloul KA. Coupled ripple oscillations between the medial temporal lobe and neocortex retrieve human memory. Science. (2019) 363:975–8. doi: 10.1126/science.aau8956, 30819961 PMC6478623

[26] Sun Y-P Wang Y-P Wang Z-H Wu F-Y Tang L-O Zhang S-W . High-frequency oscillations and the seizure onset zones in neocortical epilepsy. Chin Med J. (2015) 128:1724–7. doi: 10.4103/0366-6999.159342, 26112710 PMC4733720

[27] Zijlmans M Jiruska P Zelmann R Leijten FS Jefferys JG Gotman J. High-frequency oscillations as a new biomarker in epilepsy. Ann Neurol. (2012) 71:169–78. doi: 10.1002/ana.22548, 22367988 PMC3754947

[28] Jacobs J Zijlmans M Zelmann R Chatillon CÉ Hall J Olivier A . High-frequency electroencephalographic oscillations correlate with outcome of epilepsy surgery. Ann Neurol. (2010) 67:209–20. doi: 10.1002/ana.21847, 20225281 PMC3769290

[29] Wang S Wang IZ Bulacio JC Mosher JC Gonzalez-Martinez J Alexopoulos AV . Ripple classification helps to localize the seizure-onset zone in neocortical epilepsy. Epilepsia. (2013) 54:370–6. doi: 10.1111/j.1528-1167.2012.03721.x, 23106394

[30] Cossart R Dinocourt C Hirsch JC Merchan-Perez A De Felipe J Ben-Ari Y . Dendritic but not somatic GABAergic inhibition is decreased in experimental epilepsy. Nat Neurosci. (2001) 4:52–62. doi: 10.1038/82900, 11135645

[31] Huberfeld G Wittner L Clemenceau S Baulac M Kaila K Miles R . Perturbed chloride homeostasis and GABAergic signaling in human temporal lobe epilepsy. J Neurosci. (2007) 27:9866–73. doi: 10.1523/JNEUROSCI.2761-07.2007, 17855601 PMC6672644

[32] SwansonRA ChinigòE LevensteinD VöröslakosM MousaviN WangXJ . Topography of putative bi-directional interaction between hippocampal sharp-wave ripples and neocortical slow oscillations. Neuron. (2025) 113:754–768.e9. doi: 10.1016/j.neuron.2024.12.019, 39874961

[33] Aivar P Valero M Bellistri E de la Prida LM. Extracellular calcium controls the expression of two different forms of ripple-like hippocampal oscillations. J Neurosci. (2014) 34:2989–3004. doi: 10.1523/JNEUROSCI.2826-13.2014, 24553939 PMC6608517

[34] Avoli M De Curtis M Gnatkovsky V Gotman J Köhling R Lévesque M . Specific imbalance of excitatory/inhibitory signaling establishes seizure onset pattern in temporal lobe epilepsy. J Neurophysiol. (2016) 115:3229–37. doi: 10.1152/jn.01128.2015, 27075542 PMC4946603

[35] Bonjean M Baker T Lemieux M Timofeev I Sejnowski T Bazhenov M. Corticothalamic feedback controls sleep spindle duration in vivo. J Neurosci. (2011) 31:9124–34. doi: 10.1523/JNEUROSCI.0077-11.2011, 21697364 PMC3131502

[36] Mei N Grossberg MD Ng K Navarro KT Ellmore TM. Identifying sleep spindles with multichannel EEG and classification optimization. Comput Biol Med. (2017) 89:441–53. doi: 10.1016/j.compbiomed.2017.08.030, 28886481 PMC5650544

[37] Muehlroth BE Sander MC Fandakova Y Grandy TH Rasch B Shing YL . Precise slow oscillation–spindle coupling promotes memory consolidation in younger and older adults. Sci Rep. (2019) 9:1940. doi: 10.1038/s41598-018-36557-z, 30760741 PMC6374430

[38] Sui R Li J Shi Y Yuan S Wang H Liao J . Associations between sleep spindle metrics, age, education and executive function in young adult and middle-aged patients with obstructive sleep apnea. Nat Sci Sleep. (2024) 16:1–15. doi: 10.2147/NSS.S436824, 38213412 PMC10778138

[39] Sitnikova E. Thalamo-cortical mechanisms of sleep spindles and spike–wave discharges in rat model of absence epilepsy (a review). Epilepsy Res. (2010) 89:17–26. doi: 10.1016/j.eplepsyres.2009.09.005, 19828296

[40] Gaudreault P-O Gosselin N Lafortune M Deslauriers-Gauthier S Martin N Bouchard M . The association between white matter and sleep spindles differs in young and older individuals. Sleep. (2018) 41:zsy113. doi: 10.1093/sleep/zsy113, 29860401 PMC6132627

[41] Piantoni G Poil S-S Linkenkaer-Hansen K Verweij IM Ramautar JR Van Someren EJ . Individual differences in white matter diffusion affect sleep oscillations. J Neurosci. (2013) 33:227–33. doi: 10.1523/JNEUROSCI.2030-12.2013, 23283336 PMC6618630

[42] Fang Z Ray LB Houldin E Smith D Owen AM Fogel SM. Sleep spindle-dependent functional connectivity correlates with cognitive abilities. J Cogn Neurosci. (2020) 32:446–66. doi: 10.1162/jocn_a_01488, 31659927

[43] Schiller K Avigdor T Abdallah C Sziklas V Crane J Stefani A . Focal epilepsy disrupts spindle structure and function. Sci Rep. (2022) 12:11137. doi: 10.1038/s41598-022-15147-0, 35778434 PMC9249850

[44] Schiller K von Ellenrieder N Mansilla D Abdallah C Jaber K Garcia-Asensi A . Widespread decoupling of spindles and slow waves in temporal lobe epilepsy. Epilepsia. (2025) 66:2421–32. doi: 10.1111/epi.18359, 40085127 PMC12290998

[45] Wodeyar A Chinappen D Mylonas D Baxter B Manoach DS Eden UT . Thalamic epileptic spikes disrupt sleep spindles in patients with epileptic encephalopathy. Brain. (2024) 147:2803–16. doi: 10.1093/brain/awae119, 38650060 PMC11492493

[46] Bender AC Jaleel A Pellerin KR Moguilner S Sarkis RA Cash SS . Altered sleep microarchitecture and cognitive impairment in patients with temporal lobe epilepsy. Neurology. (2023) 101:e2376–87. doi: 10.1212/WNL.0000000000207942, 37848332 PMC10752648

[47] Kramer MA Stoyell SM Chinappen D Ostrowski LM Spencer ER Morgan AK . Focal sleep spindle deficits reveal focal thalamocortical dysfunction and predict cognitive deficits in sleep activated developmental epilepsy. J Neurosci. (2021) 41:1816–29. doi: 10.1523/JNEUROSCI.2009-20.2020, 33468567 PMC8115887

[48] Kanamori N. A spindle-like wave in the cat hippocampus: a novel vigilance level-dependent electrical activity. Brain Res. (1985) 334:180–2. doi: 10.1016/0006-8993(85)90584-0, 3995312

[49] Buzsáki G Lai-Wo SL Vanderwolf CH. Cellular bases of hippocampal EEG in the behaving rat. Brain Res Rev. (1983) 6:139–71. doi: 10.1016/0165-0173(83)90037-1 6357356

[50] Suzuki SS Smith GK. Single-cell activity and synchronous bursting in the rat hippocampus during waking behavior and sleep. Exp Neurol. (1985) 89:71–89. doi: 10.1016/0014-4886(85)90266-3, 4007117

[51] Lee AK Wilson MA. Memory of sequential experience in the hippocampus during slow wave sleep. Neuron. (2002) 36:1183–94. doi: 10.1016/S0896-6273(02)01096-6, 12495631

[52] Latchoumane CFV Ngo HVV Born J Shin HS. Thalamic spindles promote memory formation during sleep through triple phase-locking of cortical, thalamic, and hippocampal rhythms. Neuron. (2017) 95:424–435.e6. doi: 10.1016/j.neuron.2017.06.025, 28689981

[53] Geva-Sagiv M Mankin EA Eliashiv D Epstein S Cherry N Kalender G . Augmenting hippocampal–prefrontal neuronal synchrony during sleep enhances memory consolidation in humans. Nat Neurosci. (2023) 26:1100–10. doi: 10.1038/s41593-023-01324-5, 37264156 PMC10244181

[54] Maingret N Girardeau G Todorova R Goutierre M Zugaro M. Hippocampo-cortical coupling mediates memory consolidation during sleep. Nat Neurosci. (2016) 19:959–64. doi: 10.1038/nn.4304, 27182818

[55] Staresina BP Bergmann TO Bonnefond M Van Der Meij R Jensen O Deuker L . Hierarchical nesting of slow oscillations, spindles and ripples in the human hippocampus during sleep. Nat Neurosci. (2015) 18:1679–86. doi: 10.1038/nn.4119, 26389842 PMC4625581

[56] Dickey CW Verzhbinsky IA Jiang X Rosen BQ Kajfez S Eskandar EN . Cortical ripples during NREM sleep and waking in humans. J Neurosci. (2022) 42:7931–46. doi: 10.1523/JNEUROSCI.0742-22.2022, 36041852 PMC9617618

[57] Staresina BP. Coupled sleep rhythms for memory consolidation. Trends Cogn Sci. (2024) 28:339–51. doi: 10.1016/j.tics.2024.02.002, 38443198

[58] Espinal E Chrysikou EG Bickel S. Neurophysiological Mechanisms of Memory Consolidation during Sleep Uncovered through Targeted Memory Reactivation (Doctor of Philosophy). Philadelphia: Drexel University (2025).

[59] Helfrich RF Lendner JD Mander BA Guillen H Paff M Mnatsakanyan L . Bidirectional prefrontal-hippocampal dynamics organize information transfer during sleep in humans. Nat Commun. (2019) 10:3572. doi: 10.1038/s41467-019-11444-x, 31395890 PMC6687745

[60] Staresina BP Niediek J Borger V Surges R Mormann F. How coupled slow oscillations, spindles and ripples coordinate neuronal processing and communication during human sleep. Nat Neurosci. (2023) 26:1429–37. doi: 10.1038/s41593-023-01381-w, 37429914 PMC10400429

[61] Oyanedel CN Durán E Niethard N Inostroza M Born J. Temporal associations between sleep slow oscillations, spindles and ripples. Eur J Neurosci. (2020) 52:4762–78. doi: 10.1111/ejn.14906, 32654249

[62] Song I Orosz I Chervoneva I Waldman ZJ Fried I Wu C . Bimodal coupling of ripples and slower oscillations during sleep in patients with focal epilepsy. Epilepsia. (2017) 58:1972–84. doi: 10.1111/epi.13912, 28948998 PMC5669821

[63] Weiss SA Orosz I Salamon N Moy S Wei L Van ‘t Klooster MA . Ripples on spikes show increased phase-amplitude coupling in mesial temporal lobe epilepsy seizure onset zones. Epilepsia. (2016) 57:1916–30. doi: 10.1111/epi.13572, 27723936 PMC5118142

[64] Sheybani L Mégevand P Roehri N Spinelli L Kleinschmidt A van Mierlo P . Asymmetry of sleep electrophysiological markers in patients with focal epilepsy. Brain Commun. (2023) 5:fcad161. doi: 10.1093/braincomms/fcad161, 37292455 PMC10244064

[65] Clemens B Ménes A. Sleep spindle asymmetry in epileptic patients. Clin Neurophysiol. (2000) 111:2155–9. doi: 10.1016/S1388-2457(00)00482-X, 11090766

[66] Dahal P Ghani N Flinker A Dugan P Friedman D Doyle W . Interictal epileptiform discharges shape large-scale intercortical communication. Brain. (2019) 142:3502–13. doi: 10.1093/brain/awz269, 31501850 PMC6821283

[67] Ma H Wang Z Li C Chen J Wang Y. Phase–amplitude coupling and epileptogenic zone localization of frontal epilepsy based on intracranial EEG. Front Neurol. (2021) 12:718683. doi: 10.3389/fneur.2021.718683, 34566860 PMC8458805

[68] Beenhakker MP Huguenard JR. Neurons that fire together also conspire together: is normal sleep circuitry hijacked to generate epilepsy? Neuron. (2009) 62:612–32. doi: 10.1016/j.neuron.2009.05.015, 19524522 PMC2748990

[69] Gelinas JN Khodagholy D Thesen T Devinsky O Buzsáki G. Interictal epileptiform discharges induce hippocampal-cortical coupling in temporal lobe epilepsy. Nat Med. (2016) 22:641–8. doi: 10.1038/nm.4084, 27111281 PMC4899094

[70] Lambert I Roehri N Fayerstein J Giusiano B Colombet B Bénar C-G . Cortico-cortical and thalamo-cortical connectivity during non-REM and REM sleep: insights from intracranial recordings in humans. Clin Neurophysiol. (2022) 143:84–94. doi: 10.1016/j.clinph.2022.08.026, 36166901

[71] Mendes RAV Zacharias LR Ruggiero RN Leite JP Moraes MFD Lopes-Aguiar C. Hijacking of hippocampal–cortical oscillatory coupling during sleep in temporal lobe epilepsy. Epilepsy Behav. (2021) 121:106608. doi: 10.1016/j.yebeh.2019.106608, 31740330

[72] Cimbalnik J Brinkmann B Kremen V Jurak P Berry B Gompel JV . Physiological and pathological high frequency oscillations in focal epilepsy. Ann Clin Transl Neurol. (2018) 5:1062–76. doi: 10.1002/acn3.618, 30250863 PMC6144446

[73] Park CJ Hong SB. High frequency oscillations in epilepsy: detection methods and considerations in clinical application. J Epilepsy Res. (2019) 9:1–13. doi: 10.14581/jer.19001, 31482052 PMC6706641

[74] Fan Y Dong L Liu X Wang H Liu Y. Recent advances in the noninvasive detection of high-frequency oscillations in the human brain. Rev Neurosci. (2021) 32:305–21. doi: 10.1515/revneuro-2020-0073, 33661582

[75] Klinzing JG Niethard N Born J. Mechanisms of systems memory consolidation during sleep. Nat Neurosci. (2019) 22:1598–610. doi: 10.1038/s41593-019-0467-3 31451802

[76] Cai Z Sohrabpour A Jiang H Ye S Joseph B Brinkmann BH . Noninvasive high-frequency oscillations riding spikes delineates epileptogenic sources. Proc Natl Acad Sci USA. (2021) 118:e2011130118. doi: 10.1073/pnas.2011130118, 33875582 PMC8092606

[77] Liu Y Wang Y Wang T. Non-invasive localization of epileptogenic zone in drug-resistant epilepsy based on time–frequency analysis and VGG convolutional neural network. Bioengineering. (2025) 12:443. doi: 10.3390/bioengineering12050443, 40428062 PMC12109204

[78] Gerstner W. "A framework for spiking neuron models: the spike response model". In: Handbook of Biological Physics. Amsterdam: Elsevier (2001). p. 469–516.

[79] Markram H Muller E Ramaswamy S Reimann MW Abdellah M Sanchez CA . Reconstruction and simulation of neocortical microcircuitry. Cell. (2015) 163:456–92. doi: 10.1016/j.cell.2015.09.029, 26451489

[80] Breakspear M. Dynamic models of large-scale brain activity. Nat Neurosci. (2017) 20:340–52. doi: 10.1038/nn.4497, 28230845

[81] Deco G Jirsa VK Robinson PA Breakspear M Friston K. The dynamic brain: from spiking neurons to neural masses and cortical fields. PLoS Comput Biol. (2008) 4:e1000092. doi: 10.1371/journal.pcbi.1000092, 18769680 PMC2519166

[82] Wilson HR Cowan JD. Excitatory and inhibitory interactions in localized populations of model neurons. Biophys J. (1972) 12:1–24. doi: 10.1016/S0006-3495(72)86068-5, 4332108 PMC1484078

[83] Jansen BH Rit VG. Electroencephalogram and visual evoked potential generation in a mathematical model of coupled cortical columns. Biol Cybern. (1995) 73:357–66. doi: 10.1007/BF00199471, 7578475

[84] Byrne Á O’Dea RD Forrester M Ross J Coombes S. Next-generation neural mass and field modeling. J Neurophysiol. (2020) 123:726–42. doi: 10.1152/jn.00406.2019, 31774370

[85] Cona F Lacanna M Ursino M. A thalamo-cortical neural mass model for the simulation of brain rhythms during sleep. J Comput Neurosci. (2014) 37:125–48. doi: 10.1007/s10827-013-0493-1, 24402459

[86] Jajcay N Cakan C Obermayer K. Cross-frequency slow oscillation–spindle coupling in a biophysically realistic thalamocortical neural mass model. Front Comput Neurosci. (2022) 16:769860. doi: 10.3389/fncom.2022.769860, 35603132 PMC9120371

[87] Soplata AE McCarthy MM Sherfey J Lee S Purdon PL Brown EN . Thalamocortical control of propofol phase-amplitude coupling. PLoS Comput Biol. (2017) 13:e1005879. doi: 10.1371/journal.pcbi.1005879, 29227992 PMC5739502

[88] Tripathi R Gluckman BJ. Development of mechanistic neural mass (mNM) models that link physiology to mean-field dynamics. Front Netw Physiol. (2022) 2:911090. doi: 10.3389/fnetp.2022.911090, 36876035 PMC9980379

[89] Li Q Westover MB Zhang R Chu CJ. Computational evidence for a competitive thalamocortical model of spikes and spindle activity in rolandic epilepsy. Front Comput Neurosci. (2021) 15:680549. doi: 10.3389/fncom.2021.680549, 34220477 PMC8249809

[90] Pan M Li Q Song J Li D Zhang R. Spike-spindle coupling during sleep and its mechanism explanation in childhood focal epilepsy. Cogn Neurodyn. (2024) 18:2145–60. doi: 10.1007/s11571-023-10052-2, 39555302 PMC11564472

[91] Friston KJ Harrison L Penny W. Dynamic causal modelling. NeuroImage. (2003) 19:1273–302. doi: 10.1016/S1053-8119(03)00202-7, 12948688

[92] Kiebel SJ Garrido MI Moran RJ Friston KJ. Dynamic causal modelling for EEG and MEG. Cogn Neurodyn. (2008) 2:121–36. doi: 10.1007/s11571-008-9038-0, 19003479 PMC2427062

[93] Moran RJ Mallet N Litvak V Dolan RJ Magill PJ Friston KJ . Alterations in brain connectivity underlying beta oscillations in Parkinsonism. PLoS Comput Biol. (2011) 7:e1002124. doi: 10.1371/journal.pcbi.1002124, 21852943 PMC3154892

[94] Rosch RE Hunter PR Baldeweg T Friston KJ Meyer MP. Calcium imaging and dynamic causal modelling reveal brain-wide changes in effective connectivity and synaptic dynamics during epileptic seizures. PLoS Comput Biol. (2018) 14:e1006375. doi: 10.1371/journal.pcbi.1006375, 30138336 PMC6124808

[95] Gonçalves PJ Lueckmann JM Deistler M Nonnenmacher M Öcal K Bassetto G . Training deep neural density estimators to identify mechanistic models of neural dynamics. eLife. (2020) 9:e56261. doi: 10.7554/eLife.56261, 32940606 PMC7581433

